# Tocilizumab for Treatment of Severe COVID-19 Patients: Preliminary Results from SMAtteo COvid19 REgistry (SMACORE)

**DOI:** 10.3390/microorganisms8050695

**Published:** 2020-05-09

**Authors:** Marta Colaneri, Laura Bogliolo, Pietro Valsecchi, Paolo Sacchi, Valentina Zuccaro, Fabio Brandolino, Carlomaurizio Montecucco, Francesco Mojoli, Emanuele Maria Giusti, Raffaele Bruno, the COVID IRCCS San Matteo Pavia Task Force

**Affiliations:** 1 Division of Infectious Diseases I, Fondazione IRCCS Policlinico San Matteo, 27100 Pavia, Italy; marta.colaneri01@universitadipavia.it (M.C.); pietro.valsecchi01@universitadipavia.it (P.V.); p.sacchi@smatteo.pv.it (P.S.); v.zuccaro@smatteo.pv.it (V.Z.); 2 Division of Rheumatology, IRCCS Policlinico San Matteo Foundation, 27100 Pavia, Italy; l.bogliolo@smatteo.pv.it (L.B.); carlomaurizio.montecucco@unipv.it (C.M.); 3 Division of Rheumatology, IRCCS Policlinico San Matteo Foundation, University of Pavia, 27100 Pavia, Italy; fabio.brandolino01@universitadipavia.it; 4 Department of Clinical, Surgical, Diagnostic, and Paediatric Sciences, University of Pavia, 27100 Pavia, Italy; francesco.mojoli@unipv.it; 5 Anesthesia and Intensive Care, Emergency Department, Fondazione IRCCS Policlinico S. Matteo, 27100 Pavia, Italy; 6 Anesthesia, Intensive Care and Pain Therapy, Fondazione IRCCS Policlinico San Matteo, 27100 Pavia, Italy; 7 Catholic University of Milan, Department of Psychology, 20123 Milan, Italy; e.giusti@auxologico.it; 8 Istituto Auxologico Italiano IRCCS, Psychology Research Laboratory, San Giuseppe Hospital, 28824 Verbania, Italy

**Keywords:** tocilizumab, off label therapy, propensity score matching, COVID-19 pneumonia, ICU, mortality rate

## Abstract

**Objective:** This study aimed to assess the role of Tocilizumab therapy (TCZ) in terms of ICU admission and mortality rate of critically ill patients with severe COVID-19 pneumonia. **Design:** Patients with COVID-19 pneumonia were prospectively enrolled in SMAtteo COvid19 REgistry (SMACORE). A retrospective analysis of patients treated with TCZ matched using propensity score to patients treated with Standard Of Care (SOC) was conducted. **Setting:** The study was conducted at IRCCS Policlinico San Matteo Hospital, Pavia, Italy, from March 14, 2020 to March 27, 2020. **Participants:** Patients with a confirmed diagnosis of COVID-19 hospitalized in our institution at the time of TCZ availability. **Interventions:** TCZ was administered to 21 patients. The first administration was 8 mg/kg (up to a maximum 800 mg per dose) of Tocilizumab intravenously, repeated after 12 h if no side effects were reported after the first dose. **Main Outcomes and Measures:** ICU admission and 7-day mortality rate. Secondary outcomes included clinical and laboratory data. **Results:** There were 112 patients evaluated (82 were male and 30 were female, with a median age of 63.55 years). Using propensity scores, the 21 patients who received TCZ were matched to 21 patients who received SOC (a combination of hydroxychloroquine, azithromycin and prophylactic dose of low weight heparin). No adverse event was detected following TCZ administration. This study found that treatment with TCZ did not significantly affect ICU admission (OR 0.11; 95% CI between 0.00 and 3.38; p = 0.22) or 7-day mortality rate (OR 0.78; 95% CI between 0.06 and 9.34; p = 0.84) when compared with SOC. Analysis of laboratory measures showed significant interactions between time and treatment regarding C-Reactive Protein (CRP), alanine aminotransferase (ALT), platelets and international normalized ratio (INR) levels. Variation in lymphocytes count was observed over time, irrespective of treatment. **Conclusions:** TCZ administration did not reduce ICU admission or mortality rate in a cohort of 21 patients. Additional data are needed to understand the effect(s) of TCZ in treating patients diagnosed with COVID-19.

## 1. Background

At the end of December 2019, a novel coronavirus, referred to as Severe Acute Respiratory Syndrome Coronavirus 2 (SARS-CoV-2), was first reported in China as the cause of COVID-19 [1,2]. Since then, it has quickly spread worldwide, becoming a pandemic [3,4]. COVID-19 symptomatic spectrum varies from mild to critical. Pneumonia appears to be the most frequent serious manifestation of infection and approximately 6–10% of patients develop Acute Respiratory Distress Syndrome (ARDS), requiring continuous positive airway pressure therapy or mechanical ventilation [5,6].

No specific therapeutic treatment has proved successful, other than supportive care. The actual “standard of care” (SOC) includes off-label and compassionate use therapies, such as hydroxychloroquine [7], while steroid administration remains controversial [5]. Monoclonal antibodies, convalescent plasma [8] and novel antiviral drugs, as remdesivir [9] are used in selected cases. However, several drugs that have been highly promising in vitro have failed in clinical studies [10].

Although COVID-19 pathogenesis is still unclear, some patients with a severe disease have laboratory evidence of a systemic inflammation similar to cytokine release syndrome (CRS) [11,12]. CRS is characterized by a sharp increase of a large number of proinflammatory cytokines, among which IL-6 plays a pivotal role [11,12,13]. Therefore, blocking the IL-6 pathway might reduce the vigorous inflammatory response in COVID-19 [11].

Tocilizumab (TCZ) is a humanized monoclonal antibody targeting both forms of the IL-6 receptor (membrane-bound and soluble). It has been used in adults and children to treat rheumatological conditions such as rheumatoid arthritis [14] and other autoinflammatory conditions [15], or in patients with severe cytokine release syndrome (CRS) induced by chimeric antigen receptor T-cell (CAR-T) therapy [16].

Preliminary data from a single-arm Chinese trial involving 21 patients affected with severe COVID-19 treated with a single 400 mg dose of TCZ, eventually repeated after 12 h, showed clinical and radiological improvements, with a reduction in body temperature and oxygen supplementation [17].

The aim of our study is to report preliminary data from the first experience with TCZ administered to 21 patients with severe SARS-CoV-2 infection in a Regional Referral Hospital in Northern Italy. Data of TCZ treated patients are compared to those of propensity score-matched patients receiving the standard of care (SOC).

## 2. Methods

### 2.1. Participants and Study Design

The SMAtteo COvid19 REgistry (SMACORE) is the cohort of patients with confirmed diagnosis of COVID-19 disease referred to the IRCCS Policlinico San Matteo Hospital of Pavia, Italy from February 2020 The SMACORE database includes demographic, clinical (symptoms at admission and comorbidities), laboratory tests, treatment, and outcome (admission to the ICU, mortality rate, or discharge) data. Ethics approval for observational research using SMACORE data was obtained from the local ethics committee.

In this study, we report a retrospective analysis of two groups of the SMACORE cohort: patients who were administered TCZ and patients who were treated according to Standard Of Care (SOC). The SOC group included all adult patients with a confirmed SARS-CoV-2 pneumonia who were in the hospital from March 14 (the date when TCZ was available in our hospital) to March 27, 2020 treated with a combination of hydroxychloroquine (200 mg bid), azithromycin (500 mg once), prophylactic dose of low weight heparin, and methylprednisolone (a tapered dose of 1 mg/kg up to a maximum of 80 mg) for 10 days.

TCZ group included all the hospitalized adult patients with a confirmed SARS-CoV-2 pneumonia who, in addition to SOC, received TCZ from March 14, 2020 to March 27, 2020.

TCZ was administrated according to the following criteria: C-Reactive Protein (CRP) > 5 mg/dl, Procalcitonin (PCTI) < 0.5 ng/mL, arterial partial pressure of oxygen/fractional inspired oxygen (fiO2) (PF ratio) < 300 and alanine aminotransferase (ALT) < 500 U/L.

Diagnosis of SARS-CoV-2 infection was confirmed by positive Real Time Reverse Transcriptase Polymerase Chain Reaction from clinical nasal swabs, analyzed by the Molecular Virology Unit within the hospital.

To perform the analyses, we employed data collected at the day of TCZ availability and after 7 days.

### 2.2. Outcomes

Primary outcomes included admission to the ICU and 7-day mortality rate. For secondary outcomes, variations in laboratory tests, including international normalized ratio (INR), ALT, CRP, lymphocytes, neutrophils, platelets (PLT), and lactate dehydrogenase (LDH), were considered.

### 2.3. Adverse Effects

Since serious and potentially fatal infections were reported in patients receiving TCZ, patients were closely monitored for signs and symptoms of a secondary infection after TCZ administration. As hepatic injury was reported, liver function tests (LFTs) were evaluated. For the purpose of this study, we collected LFT made during the week following the date of TCZ availability.

### 2.4. Statistical Analysis

Frequencies were computed for categorical variables, whereas medians and interquartile ranges were employed to describe continuous variables.

### 2.5. Missing Data Analysis

The impact and mechanisms of missing data were inspected using frequencies and graphic methods. Patients with >65% missing data were excluded. Multiple imputation with predictive mean matching using chained equation was employed to account for the remaining missing data. Twenty datasets were created based on 30 iterations. Convergence and quality of imputed data were ascertained using graphical methods and comparing distributions of the imputed and the complete datasets. Imputed datasets were then employed to perform the subsequent analyses. To do so, models were tested in each imputed dataset and resulting parameter estimates were then pooled using a correction for degrees of freedom [18].

### 2.6. Propensity Score Matching

Nearest neighbor propensity score matching was employed to minimize differences between patients in the TCZ and SOC groups. Briefly, patients are matched case-by-case based on their prior probability of receiving TCZ. This was done by performing a regression with receiving TCZ as the dependent variable and demographic and clinical variables as predictors, assigning each patient his/her propensity score, and matching each TCZ patient with an SOC subject with a similar propensity score [19]. A one-to-one matching scheme was employed. Choice of predictors was performed in order to maximize convergence and balance of data for TCZ and SOC patients.

### 2.7. Analysis of ICU Admission and Mortality

Differences between the matched groups in ICU admission and 7-day mortality were assessed using logistic regressions. Sex and age were initially included as control variables. Due to convergence issues, sex was excluded. Then, laboratory and clinical variables were tested using univariate analyses, and only significant variables were included in the final model, along with TCZ.

### 2.8. Analysis of Secondary Outcomes

To analyze the effect of TCZ on secondary outcomes, Generalized Linear Mixed Models (GLMM) were employed. Identity link functions and normal marginal distributions were used to model changes in CRP, negative inverse link and gamma distributions in INR, lymphocytes, neutrophils and ALT, and inverse squared link and inverse gaussian distribution in LDH and PLT. To analyze the effect of TCZ administration, the factors time, intervention, and the interaction between them were included as fixed effects. Due to the presence of several outliers, data of all the secondary outcome variables were winsorized (i.e., all values beyond the 5th or 95th percentile were set to the nearest percentile).

Significance threshold was set at 0.05. Data analysis was performed mainly using R (version 3.6.1) packages *tidyverse* [20], *mice* [21], *MatchThem* [22], and *lme4* [23].

### 2.9. Patient and Public Involvement

This research was done without patient involvement. Patients were not invited to comment on the study design and were not consulted to develop patient-relevant outcomes or interpret the results. Patients were not invited to contribute to the writing or editing of this document for readability or accuracy.

## 3. Results

### 3.1. Description of the Sample and Missing Data Analysis

A total of 112 subjects were included in this analysis. Of these patients, 21 (18.75%) received TCZ + SOC, whereas 91 (81.25%) patients received SOC only. No adverse effect of TCZ was detected. Demographic and clinical characteristics of the subjects, as well as frequency of missing data are included in Table 1; Table 2.

Imputation diagnostics and density plots showed that imputation was successful and that the variables in the multiply-imputed dataset followed plausible distributions.

### 3.2. Propensity Score Matching

Variables inserted in the final propensity score matching model were sex, age, LDH, and neutrophils. All subjects were matched. Therefore, the following analyses were performed only on the 42 matched patients. Inspection of means and distributions of patients treated with TCZ and matched controls were similar. PCT was not included in the model due to convergence issues. However, all matched patients had PCT values < 0.5.

### 3.3. Effects of Tocilizumab on Mortality and ICU Admission

Logistic regressions were then performed. Regarding mortality, age and neutrophils were significant in univariate analyses. However, neutrophils were not significant when included along with age, and caused convergence issues. Neutrophils were therefore discarded and the final model included only Tocilizumab and age. The effect of Tocilizumab was not significant (Table 3).

Regarding ICU admission, age and days from hospitalization were significant in univariate analyses. The effect of Tocilizumab was not significant (Table 3).

### 3.4. Effect of Tocilizumab on Laboratory Measures

**Figure 1:**
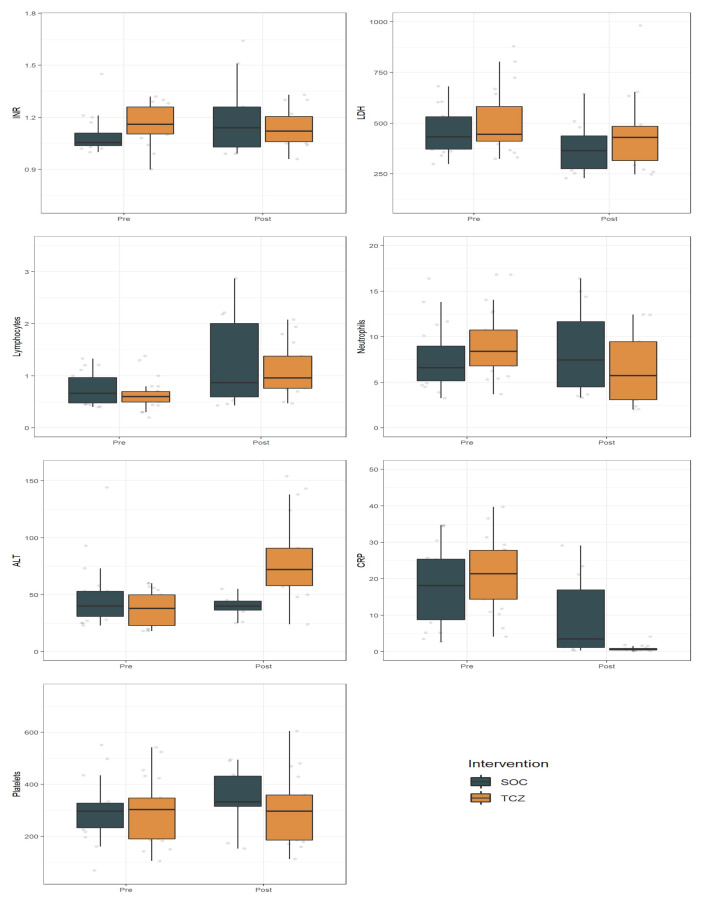
Effect of Tocilizumab (TCZ) on the laboratory measures compared with Standard of Care (SOC). Data are presented pre- and post-treatment, representing day 0 and day 7, respectively. Abbreviations: SOC, Standard of Care; INR, International Normalized Ratio; LDH, lactate dehydrogenase; ALT, alanine aminotransferase; CRP, C-Reactive Protein; PLT, platelets.

Effects of time, irrespective of treatment, were found in INR, lymphocytes, CRP, and platelets. Interactions between time and treatment were significant for INR, ALT, CRP, and platelets (Table 4, Figure 1).

## 4. Discussion

Starting from the identification of the first infected patient in Italy on 21 February 2020, the epidemic quickly spread throughout the entire country with a higher morbidity and mortality than previously observed.

In this context, physicians were faced with many seriously ill patients requiring respiratory support. This emergency required to test several off-label and compassionate-use drugs [24]. Although such an approach was justified by these unusual circumstances, it was not based on conclusive data about the efficacy and safety of proposed treatments. For this reason, randomized controlled clinical trials (RCTs) to define these objectives are urgently needed. The same position was also supported by American Thoracic Society in their recently published guidelines [25], that invite clinical researchers to produce robust data. This purpose can only be achieved by using appropriate statistical approaches in order to reduce the effects of bias and confounders.

In this study, we report preliminary data from our experience with TCZ administration in COVID-19 patients hospitalized in a Regional Referral Hospital in the Lombardy region, Italy.

Our analyses indicate that treatment with TCZ did not significantly affect ICU admission and 7-day mortality rate when compared with SOC. To date, the only studies on the effects of TCZ on mortality are a Chinese uncontrolled study which reported three deaths out of 15 patients treated with TCZ [26] and a non-peer-reviewed report on the clinical course of 21 patients, of which no one died [17].

Analysis of laboratory measures showed significant interactions between time and treatment regarding the INR, CRP, ALT, and PLT levels.

Elevated CRP levels have been a consistent finding in COVID-19 patients [26,27]. In our analysis, the striking CRP decline after treatment is an expected pharmacological effect of TCZ on the IL-6 receptor. It is to be noted that the lack of increase in CRP may be a potential problem in the diagnosis of (concurrent) infections [28].

INR was elevated at day 0, and significantly decreased over time. INR improvement in patients treated with TCZ should be considered as a positive result since an elevated INR is usually associated with more severe cases of bacterial and viral pneumonia [29].

Moreover, the lack of PLT increase in the TCZ group might reflect its effect on controlling inflammation as PLT peak has been linked to a worse prognosis of COVID-19, probably as expression of the inflammatory reaction [30].

Most patients had lymphocytopenia (defined as lymphocytes count <1.5 × 10^9^/mL) at day 0 and a significant increase at day 7 in both SOC and TCZ groups. Previous studies identified lymphocytopenia as an indicator of COVID-19 severity [31]. In our analysis, TCZ did not influence its course.

Finally, as expected, ALT increased over time in the TCZ group, but severe hepatic injuries were not observed [32].

Our analyses provide a first glimpse into the effects of TCZ in patients with COVID-19 compared to SOC. It is to be noted that patients received TCZ based on clinical and laboratory criteria that might reflect the presence of a severe disease. Thus, these results cannot be generalized to patients who are in its earlier phases. Further studies might highlight this aspect.

Currently, without approved treatments for this disease, several clinical trials are being implemented to assess potential therapies [33].

The aforementioned preliminary uncontrolled reports claimed that TCZ had positive clinical results [17] but a second trial on 15 patients treated with TCZ in combination with glucocorticoid showed controversial results, with clinical improvement in critical patients only if receiving repeated doses of TCZ [26]. Recently, two meta-analyses examining the role of IL-6 on COVID-19 [34,35] concluded that IL-6 levels are significantly elevated in patients with COVID-19 and associated with adverse clinical outcomes. So, even if preliminary data seem to be encouraging, small observational studies are subject to a number of potential problems that may bias their results.

A clinical trial in Italy (TOCIVID-19) sponsored by National Cancer Institute, Naples [36], and another one (COVACTA) sponsored by Hoffmann–La Roche [37], are now ongoing to evaluate the use of TCZ for COVID-19.

The results of several ongoing clinical trials will provide more evidence on the role of TCZ in treating COVID-19 prior to routine clinical application.

Our report has many limitations. Firstly, we chose to examine a hard clinical end point from data collected in a short period of time. Other study limitations include the small number of patients, which could have limited the power of our analyses, and the observational study design. Propensity score matching enabled us to reduce the resulting bias since it mimics randomization. However, this procedure is unable to control for the effect of variables not included in the model employed to match patients [19].

In addition, both groups received steroid therapy. Therefore, confounding influence of steroid therapy on the anti-inflammatory effects of TCZ is to be considered. Specifically, the beneficial effects of glucocorticoids in CRS [38] as well as in other extremely inflammatory conditions such as hemophagocytic lymphohistiocytosis [39], sepsis [40], or vasculitis [41] are well known.

Since the ongoing emergency, waiting for RCTs results might be tricky. Therefore, there should be a balance between gold standard research practice and the speed of implementation. This should be addressed by balancing studies with the immediate public health and clinical need for answers.

This preliminary report stems from the need to check what we have implemented, similar to a hypothetical Deming cycle model. According to this model, which consists of a logical sequence of four key steps: “P”, Plan, i.e., planning; “D”, Do, i.e., execution; “C”, Check, i.e., test and check; and “A”, Act, i.e., action (PDCA), we are actually in the “C” phase, before we complete the intervention.

These data will allow us to make any correction to the planned intervention.

## 5. Conclusions

In conclusion, this preliminary analysis suggests that TCZ administration did not reduce ICU admission and mortality rate. Conversely, while some laboratory markers improved in patients treated with TCZ, ALT increased.

Should TCZ be considered as a useless option in COVID-19 pandemic? Obviously not, since current clinical data on TCZ effectiveness are lacking. In order to promptly share our experience with other clinicians currently involved in this scenario, we reported our preliminary results.

Additional data are needed to understand the effect(s) of TCZ in treating critically ill patients diagnosed with COVID-19.

## Figures and Tables

**Table 1:** Frequencies of demographic and clinical characteristics of the SMACORE cohort.

		Whole Sample		Stratified by Treatment	
		(*n* = 112)		SOC (*n* = 91)	Tocilizumab (*n* = 21)
		n	% Missing	n	n
Sex	Male	82	0	63	19
	Female	30		28	2
Death day 7	Yes	24	0	19	5
	No	88		72	16
ICU admission day 7	Yes	15	0	12	3
	No	97		79	18
Interstitial lung disease day 0	Yes	53	49.1	41	12
	No	4		3	1
Past tumor	Yes	4	50	3	1
	No	52		40	12
Heart diseases	Yes	9	50	7	2
	No	47		36	11
Hypertension	Yes	28	50	20	8
	No	28		23	5
Diabetes	Yes	10	50	8	2
	No	46		35	11
Lung diseases	Yes	4	50	4	0
	No	52		39	13
Obesity	Yes	16	50	12	4
	No	40		31	9
Other comorbidities	Yes	16	50	12	4
	No	40		31	9

Abbreviation: SOC, Standard of Care.

**Table 2:** Bivariate analysis of laboratory measures in the whole sample and stratified by treatment.

	Whole Sample			Stratified by Treatment			
				SOC		Tocilizumab	
	Median	IQR	Missing %	Median	IQR	Median	IQR
Age (y)	63.55	16.95	0.00	63.74	16.32	62.33	18.68
Days of hospitalization	14	5.25	0	14.00	4.00	2.00	6.00
INR day 0	1.11	0.16	16.07	1.09	0.15	1.16	0.16
INR day 7	1.17	0.21	55.35	1.20	0.26	1.12	0.15
LDH, 100 U/L day 0	441	219	9	439	228	445	172
LDH, 100 U/L day 7	414	228	48	397	237	430	169
Lymphocytes, 10^9^/mL day 0	0.74	0.50	4.46	0.80	0.50	0.60	0.20
Lymphocytes, 10^9^/mL day 7	0.93	0.69	42.86	0.90	0.80	0.96	0.62
Neutrophils, 10^9^/mL day 0	6.43	4.34	4.46	6.08	4.02	8.40	3.94
Neutrophils, 10^9^/mL day 7	7.25	6.73	42.86	7.44	6.70	5.73	6.37
ALT, U/L day 0	41	34	6.25	43.00	38.75	38.00	27.00
ALT, U/L day 7	56	53.25	46.43	40.00	44.50	72.00	33.00
CRP, mg/L day 0	15.61	13.75	3.57	14.88	14.41	21.38	13.40
CRP, mg/L day 7	2.37	14.02	40.18	6.07	16.42	0.63	0.45
PCT, ng/mL day 0	0.27	0.81	11.61	0.31	1.37	0.24	0.14
PLT, 10^9^/mL day 0	270	141	4.46	252.50	139.75	303.00	157.00
PLT, 10^9^/mL day 7	310	139.50	42.86	313	128.50	296	174.00
P/F ratio day 0	197.5	194.33	60.71	144.00	222.05	224.80	62.00

Abbreviations: SOC, Standard of Care; IQR, Interquartile Range; INR, International Normalized Ratio; LDH, lactate dehydrogenase; ALT, alanine aminotransferase; CRP, C-Reactive Protein; PCT, procalcitonin PLT, platelets; P/F ratio, indicator of respiratory failure.

**Table 3:** Logistic regression to evaluate the effect of Tocilizumab on mortality and ICU admission.

	Mortality						ICU Admission					
	Estimate (SE)	Statistic	DF	*p*	OR	95% CI	Estimate (SE)	Statistic	DF	*p*	OR	95% CI
Intercept	−15.63 (6.21)	−2.52	29.19	0.02	0	[0.00, 0.03]	−1.90 (3.00)	−0.63	3.22	0.53	0.15	[0.00, 53.80]
Age	0.21 (.09)	2.43	28.54	0.02	1.24	[1.04, 1.47]	−0.06 (.04)	−1.35	30.09	0.18	0.94	[0.86,1.02]
Days of hospitalization							0.25 (.12)	2.08	33.32	0.04	1.29	[1.01, 1.64]
TCZ	−0.25 (1.27)	−0.2	24.18	0.84	0.78	[0.06, 9.34]	−2.18 (1.74)	−1.26	29.54	0.22	0.11	[0.00, 3.38]

Estimates are unstandardized. Abbreviations: SE, Standard Errors; DF, Degrees of Freedom; OR, Odds Ratios; CI, Confidence Intervals; TCZ, Tocilizumab administration.

**Table 4:** Effect of Tocilizumab on laboratory measures.

	INR			LDH			Lymphocytes			Neutrophils			ALT			CRP			PLT		
	Est	SE	p	Est	SE	p	Est	SE	p	Est	SE	p	Est	SE	p	Est	SE	p	Est	SE	p
Intercept	0.92	0.04	0.00	486.59	42.89	0.00	1.62	0.17	0.00	7.92	2.10	0.00	47.78	22.01	0.03	18.23	2.25	0.00	322.60	32.11	0.00
Time	−0.09	0.04	0.03	−75.89	63.24	0.24	−0.34	0.13	0.01	0.44	3.23	0.89	48.01	34.67	0.17	−8.45	2.83	0.00	−3.32	44.32	0.94
TCZ	−0.09	0.05	0.09	25.30	58.81	0.67	0.13	0.22	0.57	1.27	2.97	0.67	−11.02	31.10	0.72	2.56	3.16	0.42	−17.55	44.90	0.70
Time × TCZ	0.17	0.05	0.00	22.80	86.15	0.79	−0.16	0.18	0.36	1.69	4.54	0.71	−9.30	46.82	0.84	−7.84	3.60	0.03	1.23	62.51	0.98

Abbreviations: Est, Unstandardized Estimates; SE, Standard Errors; INR, International Normalized Ratio; LDH, lactate dehydrogenase; ALT, alanine aminotransferase; CRP, C-Reactive Protein; PLT, platelets; TCZ, Tocilizumab administration.
